# Associations of migrant and refugee status with physical comorbidity and mortality among young adults with incident non-affective psychotic disorders

**DOI:** 10.1186/s12889-025-24054-8

**Published:** 2025-08-06

**Authors:** Alexis E. Cullen, Christopher J. de Montgomery, Emma Pettersson, Aemal Akhtar, Marie Norredam, Heidi Taipale, Ellenor Mittendorfer-Rutz

**Affiliations:** 1 Department of Clinical Neuroscience, Division of Insurance Medicine, Karolinska Institutet, Berzelius vag 3, Stockholm, 171 77 Sweden; 2 Department of Psychosis Studies, Institute of Psychiatry, Psychology & Neuroscience, King’s College London, London, UK; 3 Department of Public Health, Danish Research Centre for Migration, Ethnicity and Health (MESU), University of Copenhagen, Copenhagen, Denmark; 4 Section of Immigrant Medicine, Department of Infectious Diseases, University Hospital Hvidovre, Copenhagen, Denmark; 5 Niuvanniemi Hospital, Kuopio, Finland; 6 School of Pharmacy, University of Eastern Finland, Kuopio, Finland

**Keywords:** Schizophrenia, Multimorbidity, Somatic disorders, Health inequalities, Immigration

## Abstract

**Background:**

Whilst a ‘healthy migrant effect’ has been reported for some migrant groups, it is unclear whether this generalises to individuals with non-affective psychotic disorders (NAPDs), a population characterised by increased risk of physical morbidity and mortality. We aimed to compare the risk of developing physical conditions, all-cause mortality, and suicide among Swedish-born, non-refugee migrant, and refugee individuals with incident NAPDs.

**Method:**

In this register-based cohort study, nationwide registers were used to identify individuals aged 18–35 years who received their first diagnosis of NAPD in inpatient/specialist outpatient care between 1 January 2006 and 31 December 2013. Individuals were followed from the date of cohort entry until 31 December 2018 or death/emigration. Cox proportional hazards models (yielding hazard ratios, HRs and 95% confidence intervals, CI), incorporating inverse-probability weights to account for covariate differences across population groups, were used to compare the risk of specific physical disorders, all-cause mortality, and suicide in refugees and non-refugee migrants to their Swedish-born peers.

**Results:**

We identified 7,733 individuals (median age 26, IQR 22–30 years; 63.6% male) with incident NAPDs. Compared to their Swedish-born peers, the risk of developing type 2 diabetes was significantly increased among refugees (HR = 2.48, 95% CI = 1.56–3.95) and non-refugee migrants (HR = 1.66, 95% CI = 1.01–2.71) with NAPDs, refugees were also at higher risk of infectious diseases (HR = 1.30, 95% CI = 1.09–1.55). No significant differences in cardiovascular and respiratory diseases were observed. In contrast, refugees with NAPDs had lower risk of all-cause mortality (HR = 0.59, 95% CI = 0.39 − 0.91) and deaths due to suicide (HR = 0.57, 95% CI = 0.34 − 0.96) compared to Swedish-born individuals with NAPDs.

**Conclusion:**

Among those with NAPDs, migrants (particularly refugees) are at greater risk of developing diabetes and infectious diseases. Given the global increase in refugee populations, our findings have important implications for healthcare providers and suggest that these vulnerable individuals may require closer monitoring of physical health.

**Supplementary Information:**

The online version contains supplementary material available at 10.1186/s12889-025-24054-8.

## Background

Schizophrenia is associated with an increased risk of mortality compared to the general population [1, 2]. This elevation in risk is evident even among individuals who are in the early phases of illness, and leads to an estimated 10-25-year reduction in life expectancy [3]. Whilst the excess mortality among individuals with psychosis is largely attributable to suicide, deaths due to physical disorders are also elevated relative to the general population [1]. Consistent with this finding, several physical disorders, including infectious, cardiovascular, musculoskeletal, respiratory, nervous system, endocrine, metabolic, and autoimmune diseases [4–6] are more common among individuals with schizophrenia and other non-affective psychotic disorders (NAPDs). Moreover, physical multimorbidity (defined as experiencing 2 or more chronic physical disorders) is estimated to occur in as many as 2 in 5 individuals with NAPDs [7]. Several potentially-modifiable environmental factors such as antipsychotic medication, tobacco and substance use, poor diet, and barriers to accessing healthcare, have been hypothesised to explain these findings [1, 5–8]. However, genetic factors and a family history of illness may contribute to the increased risk of some specific disorders (e.g., autoimmune disorders and type 2 diabetes) among individuals with NAPDs [6, 9]. Irrespective of the underlying mechanisms, which are likely multifactorial, determining whether there are subgroups of individuals with NAPDs who are more likely to experience excess morbidity and mortality is necessary to ensure that interventions to monitor and improve physical health are directed to those with the greatest need.

Consistent with the finding that psychiatric disorders are highly prevalent among migrants [10, 11], particularly refugees [10, 12, 13], meta-analyses show that non-refugee migrants and refugees are at greater risk of developing NAPDs compared to the general population [14–18]. Many of the factors that likely increase the risk of psychiatric disorders among migrants (e.g., underutilisation of healthcare, socioeconomic disadvantage, and psychosocial stressors) could plausibly lead to poorer physical health and mortality in these groups. Some studies have shown that within the general population, migrants (including refugees) have lower rates of specific physical disorders and all-cause mortality compared to the host population [19–22]. Such findings, referred to as the ‘healthy migrant effect’, have been attributed to both internal and external selection biases (i.e., healthier individuals having more opportunities and motivation to migrate, whilst also being more likely to be granted residency) and protective cultural factors [23]. However, more recent evidence suggests that not all migrant groups experience this protective effect. Indeed, other studies have found that that some non-refugee migrants and refugees experience poorer physical health and higher mortality rates, with region of origin, level of education, duration of residency, and the presence of specific psychiatric disorders appearing to moderate these effects [23–25]. With regard to differences in physical health outcomes among individuals with psychotic disorders, a recent review found no studies examining the impact of migrant/refugee status on physical morbidity among individuals with first-episode psychosis [26]. We are aware of only one study examining all-cause mortality, which found that refugees with psychotic disorders, but not non-refugee migrants, had higher mortality rates than Swedish-born individuals with psychosis [27]. Studies investigating suicide have yielded inconsistent findings: a large study from the Netherlands found that deaths due to suicide were less common among first-generation migrants with NAPDs compared to native-born individuals with NAPDs [28], whilst a Swedish study observed similar rates of suicide in native-born individuals with NAPDs and refugees with NAPDs after adjustment for confounders [29]. However, the divergence in findings may reflect the fact that these studies examined different migrant groups (i.e., all migrants vs. refugees only).

The present study aimed to address these knowledge gaps by comparing the risk of physical disorders, all-cause mortality, and suicide among Swedish-born, non-refugee migrant, and refugee groups with NAPDs accounting for group differences in sociodemographic, work incapacity, and clinical factors at baseline. We used data from a well-characterised cohort of individuals aged 18–35 years who were treated for their first episode of NAPD in Sweden between 2006 and 2013 [30, 31]. The young age range of this cohort makes it ideal for identifying morbidity and mortality risks which are potentially more modifiable. Given the inconsistent findings from the few studies examining the impact of migrant/refugee status on all-cause mortality and suicide among individuals with psychotic disorders, we had no hypotheses regarding the direction of differences for these outcomes. Our previous studies using data from this cohort showed that refugees and non-refugee migrants with NAPDs spend more time in hospital and are more likely to be unemployed compared to their native-born peers [30, 31], potentially increasing exposure to infections/high doses of antipsychotics and economic disadvantage, respectively. We therefore hypothesised that migrant groups would be at greater risk of specific physical disorders.

## Method

### Study design and setting

This prospective cohort study used data from the Swedish national registers (described below) linked by the (pseudonymised) unique personal identification number assigned to all Swedish residents at birth or immigration. According to current Swedish regulations, the use of register data for research purposes does not require informed consent from individuals held in these registers. All procedures were approved by the Regional Ethical Review Board in Sweden (2007/762 − 31 and 2016-1533-32). This article follows the Strengthening the Reporting of Observational Studies in Epidemiology (STROBE) reporting guideline.

### Study population

The Swedish National Patient Register (NPR, maintained by the National Board of Health and Welfare [32, 33]) which has captured all inpatient and specialist outpatient care since 1987 and 2001, respectively, was used to define the study population. Individuals were identified according to International Classification of Diseases– version 10 (ICD-10 [34]) codes assigned at discharge/physician contact (for inpatient/outpatient treatments, respectively). We first identified all individuals aged 18–35 years in Sweden who received their first main diagnosis of NAPD (ICD-10 codes F20-F29) in inpatient/specialist outpatient care between the 1 st of January 2006 and 31 st of December 2013. To capture incident NAPD cases, individuals were required to have no recorded inpatient/outpatient contacts with a main diagnosis of NAPD in the previous three years (1080 days) and to have resided in Sweden for at least three full calendar years prior to cohort entry (as determined using the Longitudinal Integrated Database for Health Insurance and Labour Market Studies, LISA [35], maintained by Statistics Sweden). In addition, data from the Prescribed Drug Register (PDR, administered by the National Board of Health and Welfare [36]) were used to exclude individuals who had any recorded purchases of antipsychotic medication (Anatomical Therapeutic Chemical [ATC] classification codes N05A, omitting lithium N05AN) in the 15 − 3 months prior to cohort entry. Individuals entered the cohort on the date of their first contact for NAPD (between 2006 and 2013) and were followed until 31 December 2018 (minimum of 5 years) or until death/emigration.

### Exposure

Exposure status was determined using data from LISA and the Longitudinal Database for Integration Studies register (STATIV, developed by Statistics Sweden and the Swedish Integration Board [37]). Individuals whose grounds for residence was registered as “refugee status” or “family reunification with a refugee” were classified as refugees, all other individuals born abroad were classified as non-refugee migrants. The native-born group comprised all individuals born in Sweden.

### Outcomes

We selected four physical health outcomes which are highly prevalent among individuals with psychotic disorders [4, 38–40], namely cardiovascular diseases (ICD-10 codes: G45, I20-I25, I47-I50, I60-I69), type 2 diabetes mellitus (ICD-10 code: E11), respiratory diseases (ICD-10 codes: J42, J44, J12-J18^1^), and infectious diseases (ICD-10 codes: A00-B99, J00-J18, K35, L00-L08, M00, N70–N72, N76, O23, O264, O85–O86, O98). These outcomes were determined during the follow-up period (ranging from 5 to 13 years depending on year of cohort entry) using the NPR and defined as any inpatient/specialist outpatient treatment with corresponding ICD-10 codes recorded as main or side diagnoses. Death during the follow-up period was determined using the using the Cause of Death Register (National Board of Health and Welfare [41]). Statistical analyses were performed for all-cause mortality and deaths attributable to suicide (ICD-10 codes: X60-X84, Y10-Y34).

### Covariates

Sociodemographic variables were obtained from the LISA database. Age in years was measured during the year of cohort entry; gender, level of education (compulsory, 0–9 years vs. high school, 10–12 years vs. university, > 12 years), family situation (single vs. married/cohabiting), type of residence region (cities vs. towns/suburbs vs. rural areas), household income (categorised as annual income ≥60th percentile of the median that year vs. annual income < 60th percentile of the median) and unemployment days (none vs. any) were measured on the 31 st December in the calendar year prior to cohort entry. Sickness absence ( ≤30 days vs. >30 days) and disability pension (none vs. any), indexing functional impairment associated with psychiatric and/or physical disorders, were obtained for the calendar year prior to cohort entry using data from the Micro-Data for Analyses of Social Insurance (MiDAS, Swedish Social Insurance Agency [42]). NAPD diagnosis at first contact (schizophrenia, ICD-10: F20 vs. schizotypal disorder, ICD-10 F21 vs. persistent delusional disorder, ICD-10: F22 vs. acute and transient psychotic disorder, ICD-10: F23 vs. other, ICD-10: F24-F29) and prior treatment for other (non-psychotic) psychiatric disorders (ICD-10 F codes excluding F20-F29), physical disorders (ICD-10 codes A-Z, excluding F, O80, X60-X84, Y10-Y34, and Z), and suicide attempts (ICD-10 codes X60-X84, Y10-Y34) during the three years (1080 days) prior to cohort entry were determined using the NPR. Dispensations of any psychotropic medications (no vs. yes), including antipsychotics, lithium, anxiolytics, benzodiazepines, hypnotics, Z drugs, antidepressants, and mood stabilisers (ATC codes N05A, N05B, N05C, N06A, N03AF01, N03AG01, N03AX09, and N05AN01) during the six months (180 days) prior to cohort entry were determined using the PDR.

### Statistical methods

Inverse probability weighted Cox proportional hazards models were used to estimate the marginal effect of population group status on the hazard of developing each physical health outcome (cardiovascular disease, type 2 diabetes, infectious diseases, and respiratory diseases), all-cause mortality, and suicide. Outcomes were examined in separate Cox models in which individuals who had received a diagnosis of that disease in the three years (1080 days) prior to cohort entry were excluded from the model, resulting in different sample sizes for each analysis. In each analysis, inverse probability weighting (IPW) was used to account for potential confounders (all covariates listed above) by balancing the distribution of covariates across population groups. The advantage of this approach over multivariable regression is that potential confounders are summarised as a single variable, which is useful when there are a large number of covariates and/or a small number of events [43]. Several methods for generating weights were considered including multinomial logistic regression, parametric and nonparametric covariate balancing propensity score weighting, generalized boosted models, optimization-based weighting, and entropy balancing. Weights produced using entropy balancing were selected as the most efficient based on balance metrics (standardised mean differences and weighted proportion differences for continuous and categorical covariates, respectively) and the resulting effective (weighted) sample sizes. This technique aims to minimise the entropy distance between the covariate distributions across exposure groups, and has been shown to reduce confounding and produce more effective weights compared to other methods such as logistic regression [44]. Generated weights were then incorporated into Cox proportional hazards models; observations were censored at death/emigration. We present the hazard ratios (HR) and associated 95% confidence intervals (CI) for non-refugee migrant and refugee groups relative to the Swedish-born group and Kaplan Meier survival curves incorporating IPWs. Statistical analyses were performed in R (version 4.3.1) utilising the ‘survival’, ‘weightit’, ‘ebal’ and ‘cobalt’ packages.

## Results

### Sample characteristics

In total, 7,733 individuals (75.9%, *n* = 5,870 native-born; 11.1%, *n* = 861 non-refugee migrants; 13.0%, *n* = 1,002 refugees) received their first diagnosis of NAPD between 2006 and 2013 and were followed for a mean ± S.D. follow-up time 8.60 ± 2.79 years (see Supplementary Table 1 for follow-up time by population group). Europe was the most common region of birth among both non-refugee migrants and refugees and the majority had resided in Sweden for 11 or more years (see Supplementary Table 2).

Characteristics of the crude (unweighted) sample are provided by population group in Table 1. There were notable differences in sociodemographic factors across the groups. Non-refugee migrants were somewhat older at first diagnosis (median, IQR age: 29, 24–32 years) than both Swedish-born (median, IQR age: 25, 21–30 years) and refugee groups (median, IQR age: 25, 22–29 years) whilst refugees were more likely to be male (70.6% of refugees vs. 63.7% of Swedish-born and 54.7% of non-refugee migrants). Compared with Swedish-born individuals, non-refugee migrant and refugee groups were more likely to be living in a low-income household, residing in a city, and to have been unemployed in the year prior to first diagnosis. Non-refugee migrants, however, were most likely to have completed university education. At first contact, ‘other’ psychotic disorders (ICD-10 codes: F24-F29) were the most common diagnosis, with no marked differences across groups. The proportion of individuals who had received previous secondary care treatment for any other (non-psychotic) psychiatric disorder was highest among Swedish-born individuals (53.0% vs. 42.0% and 45.0% in non-refugee migrants and refugees, respectively) as was use of any psychotropic medication in the 6 months prior to cohort entry.

**Table 1:** Baseline characteristics of individuals diagnosed with incident non-affective psychotic disorders by population group

	Total cohort		Swedish-born		Non-refugee migrant		Refugee	
	*N*	(%)	*N*	(%)	*N*	(%)	*N*	(%)
Total (row percentage)	7733	100	5870	75.9	861	11.1	1002	13.0
Age (years), median (IQR)^a^	26	(22–30)	25	(21–30)	29	(24–32)	25	(22–29)
Gender^b^								
Men	4919	(63.6)	3741	(63.7)	471	(54.7)	707	(70.6)
Women	2814	(36.4)	2129	(36.3)	390	(45.3)	295	(29.4)
Level of education^b^								
Compulsory	3903	(50.5)	2793	(47.6)	462	(53.7)	648	(64.7)
High school	2322	(30.0)	1899	(32.4)	188	(21.8)	235	(23.5)
University	1508	(19.5)	1178	(20.1)	211	(24.5)	119	(11.9)
Family situation^b^								
Single	7171	(92.7)	5637	(96.0)	681	(79.1)	853	(85.1)
Married/cohabiting	562	(7.3)	233	(4.0)	180	(20.9)	149	(14.9)
Type of residence region^b^								
Cities	3678	(47.6)	2594	(44.2)	491	(57.0)	593	(59.2)
Towns/suburbs	2879	(37.2)	2283	(38.9)	282	(32.8)	314	(31.3)
Rural areas	1176	(15.2)	993	(16.9)	88	(10.2)	95	(9.5)
Household income^b^								
>=60 pct of median	4572	(59.1)	3676	(62.6)	432	(50.2)	464	(46.3)
< 60 pct of median	3161	(40.9)	2194	(37.4)	429	(49.8)	538	(53.7)
Unemployment days^b^								
None	5504	(71.2)	4328	(73.7)	574	(66.7)	602	(60.1)
Any	2229	(28.8)	1542	(26.3)	287	(33.3)	400	(39.9)
Sickness absence days^b^								
<= 30	7230	(93.5)	5448	(92.8)	824	(95.7)	958	(95.6)
> 30	503	(6.5)	422	(7.2)	37	(4.3)	44	(4.4)
Disability pension receipt^b^								
None	6788	(87.8)	5096	(86.8)	776	(90.1)	916	(91.4)
Any	945	(12.2)	774	(13.2)	85	(9.9)	86	(8.6)
Year of cohort entry								
2006	962	(12.4)	740	(12.6)	120	(13.9)	102	(10.2)
2007	880	(11.4)	649	(11.1)	108	(12.5)	123	(12.3)
2008	947	(12.2)	727	(12.4)	98	(11.4)	122	(12.2)
2009	957	(12.4)	736	(12.5)	92	(10.7)	129	(12.9)
2010	950	(12.3)	710	(12.1)	107	(12.4)	133	(13.3)
2011	969	(12.5)	756	(12.9)	106	(12.3)	107	(10.7)
2012	1015	(13.1)	749	(12.8)	112	(13.0)	154	(15.4)
2013	1053	(13.6)	803	(13.7)	118	(13.7)	132	(13.2)
NAPD diagnosis at first contact								
Schizophrenia	634	(8.2)	464	(7.9)	67	(7.8)	103	(10.3)
Schizotypal	139	(1.8)	129	(2.2)	< 10	(NR)	< 10	(NR)
Delusional disorder	675	(8.7)	505	(8.6)	> 70	(NR)	> 70	(NR)
Acute or transient	2663	(34.4)	2050	(34.9)	294	(34.1)	319	(31.8)
Other	3622	(46.8)	2722	(46.4)	410	(47.6)	490	(48.9)
Prior psychiatric disorder^c^								
None	3806	(49.2)	2756	(47.0)	499	(58.0)	551	(55.0)
Any	3927	(50.8)	3114	(53.0)	362	(42.0)	451	(45.0)
Prior physical condition^c^								
None	3218	(41.6)	2458	(41.9)	362	(42.0)	398	(39.7)
Any	4515	(58.4)	3412	(58.1)	499	(58.0)	604	(60.3)
Prior suicide attempt^c^								
None	7224	(93.4)	5452	(92.9)	815	(94.7)	957	(95.5)
Any	509	(6.6)	418	(7.1)	46	(5.3)	45	(4.5)
Psychotropic medication^d^								
None	4948	(64.0)	3596	(61.3)	606	(70.4)	746	(74.5)
Any	2785	(36.0)	2274	(38.7)	255	(29.6)	256	(25.5)

NR not reported (data suppressed to prevent determination of cells with counts less than 10)

*IQR* interquartile range, *NAPD* non-affective psychotic disorder

^a^Measured during year of cohort entry

^b^Measured on 31 st December in the calendar year prior to cohort entry

^c^Measured in the three relative years (1080 days) prior to cohort entry date

^d^Measured in the 6 months (180 days) prior to cohort entry date

### Inverse probability weighting

For each outcome analysis, IPWs were used to balance the distribution of covariates across population groups, resulting in five separate weighted samples (sample sizes for each outcome differed due to the exclusion of individuals who experienced these outcomes prior to cohort entry; thus, the sample sizes for all-cause mortality and suicide were the same). To assess the extent to which weighting was effective, we derived standardised mean differences and weighted proportion differences (for continuous and categorical covariates, respectively) in the crude and weighted samples for all pairwise comparisons (non-refugee native vs. native-born, refugee vs. native-born, and refugee vs. non-refugee migrant). All maximum difference values were reduced to  ≤0.0001 in the weighted samples (see Supplementary Table 3) indicating that population groups within the five samples were balanced on all covariates.

### Physical disorders

Rates of cardiovascular disease, type 2 diabetes, infectious disease, and respiratory disease during the follow-up period within the crude (unadjusted) sample and weighted (effective) sample are provided in Table 2. Across all population groups, infectious disease was the most common outcome, with event rates in the weighted sample ranging from 9.22 per 100,000 among Swedish-born individuals to 12.08 per 100,000 among refugees. Rates of all other diseases, especially cardiovascular diseases, were markedly lower (ranging from 0.53 to 1.41 per 100,000).

**Table 2:** Risk of specific physical disorders, all-cause mortality, and suicide among individuals diagnosed with incident non-affective psychotic disorders during the 5–13-year follow-up by population group

Model^a^	Unadjusted sample^b^			Weighted sample^c^			Cox regression model^d^		
	Sample size	Number of events	Event rate (per 100,000 person-years)	Effective sample size	Number of events	Event rate (per 100,000 person-years)	HR	95% CI	*p*
Cardiovascular disease									
Swedish-born	5827	106	(0.59)	5509	105.36	(0.59)	Ref	—	
Non-refugee migrant	854	15	(0.59)	604	15.57	(0.60)	1.03	0.56–1.87	0.930
Refugee	997	19	(0.63)	623	16.15	(0.53)	0.90	0.53–1.55	0.715
Type 2 Diabetes									
Swedish-born	5861	100	(0.55)	5542	102.66	(0.57)	Ref	—	
Non-refugee migrant	856	27	(1.06)	601	24.25	(0.94)	**1.66**	**1.01–2.71**	**0.045**
Refugee	998	38	(1.26)	622	42.51	(1.41)	**2.48**	**1.56–3.95**	**< 0.001**
Infectious diseases									
Swedish-born	4891	1207	(9.25)	4638	1201.46	(9.22)	Ref	—	
Non-refugee migrant	707	174	(9.56)	499	167.55	(9.01)	0.98	0.81–1.17	0.794
Refugee	847	238	(11.13)	540	254.48	(12.08)	**1.30**	**1.09–1.55**	**0.003**
Respiratory disease									
Swedish-born	5827	161	(0.90)	5510	165.54	(0.93)	Ref	—	
Non-refugee migrant	855	23	(0.91)	603	25.93	(1.01)	1.09	0.65–1.82	0.747
Refugee	996	36	(1.20)	624	37.86	(1.26)	1.35	0.90–2.04	0.147
All-cause mortality									
Swedish-born	5870	340	(1.86)	5548	340.42	(1.86)	Ref	—	
Non-refugee migrant	861	33	(1.27)	606	34.39	(1.31)	0.70	0.47–1.04	0.076
Refugee	1002	34	(1.11)	626	34.31	(1.11)	**0.59**	**0.39–0.91**	**0.016**
Suicide									
Swedish-born	5870	190	(1.04)	5548	189.92	(1.04)	Ref	—	
Non-refugee migrant	861	20	(0.77)	606	20.07	(0.76)	0.73	0.44–1.22	0.228
Refugee	1002	21	(0.68)	626	18.36	(0.59)	**0.57**	**0.34–0.96**	**0.035**

*HR* hazard ratio, *CI* confidence interval

^a^Each outcome (cardiovascular disease, type 2 diabetes, infectious diseases, respiratory diseases, all-cause mortality, suicide) examined in a separate Cox regression model

^b^For each physical disorder, the unadjusted sample excludes individuals previously treated for the condition in the three years (1080 days) prior to the first diagnosis of non-affective psychotic disorder

^c^Weighted sample derived using inverse probability weighting to account for population group differences in covariates at baseline

^d^Cox regression model incorporates inverse probability weights, bold font indicates statistical significance at the *p*<0.05 level

**Fig. 1:**
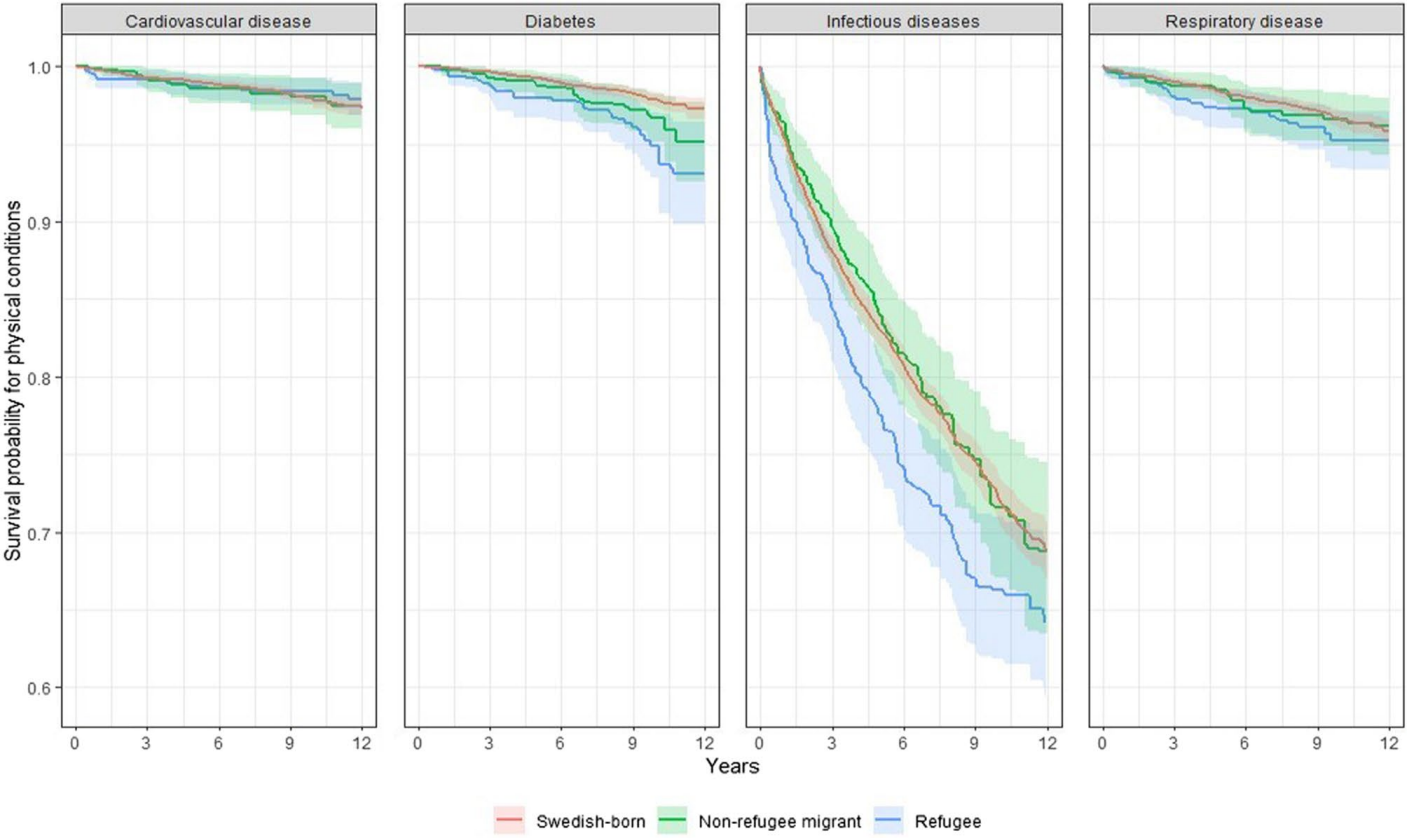
Kaplan-Meier estimated survival curves, incorporating inverse probability weights, for physical disorders during the 5–13-year follow-up period among Swedish-born, non-refugee migrant, and refugee groups with non-affective psychotic disorders

Cox proportional hazards models (applied to weighted samples) showed that the risk of developing type 2 diabetes compared to Swedish-born individuals was significantly higher among both non-refugee migrants (HR = 1.66; 95% CI = 1.01 to 2.71) and refugees (HR = 2.48; 95% CI = 1.56 to 3.95). Refugees were also at higher risk of experiencing infectious diseases when compared to their Swedish-born peers (HR = 1.30; 95% CI = 1.09 to 1.55). No significant differences in risk of cardiovascular or respiratory diseases were observed across populations (Table 2; Fig. 1).

### All-cause mortality and suicide

**Fig. 2:**
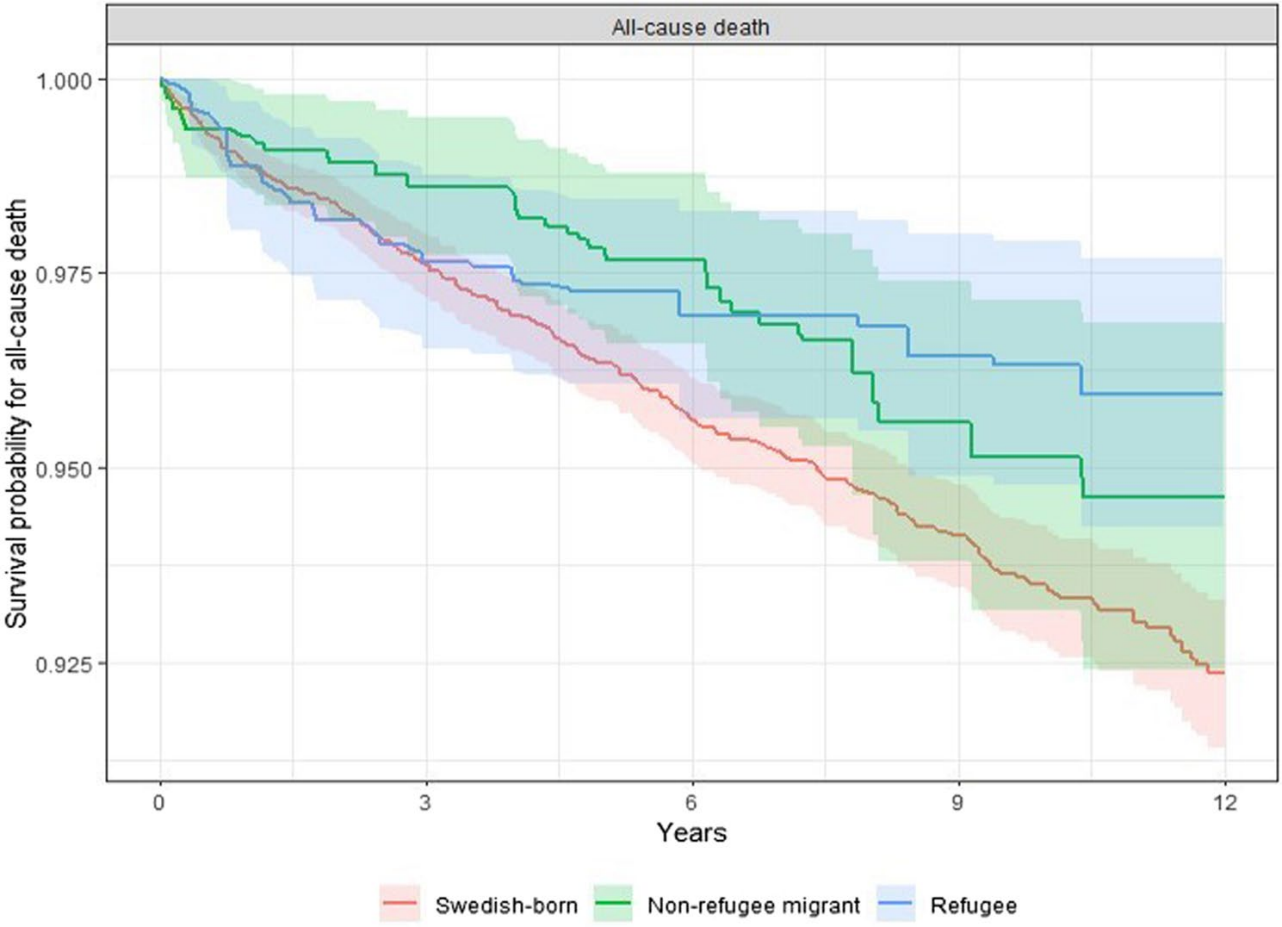
Kaplan-Meier estimated survival curves, incorporating inverse probability weights, for all-cause mortality during the 5–13-year follow-up period among Swedish-born, non-refugee migrant, and refugee groups with non-affective psychotic disorders

**Fig. 3:**
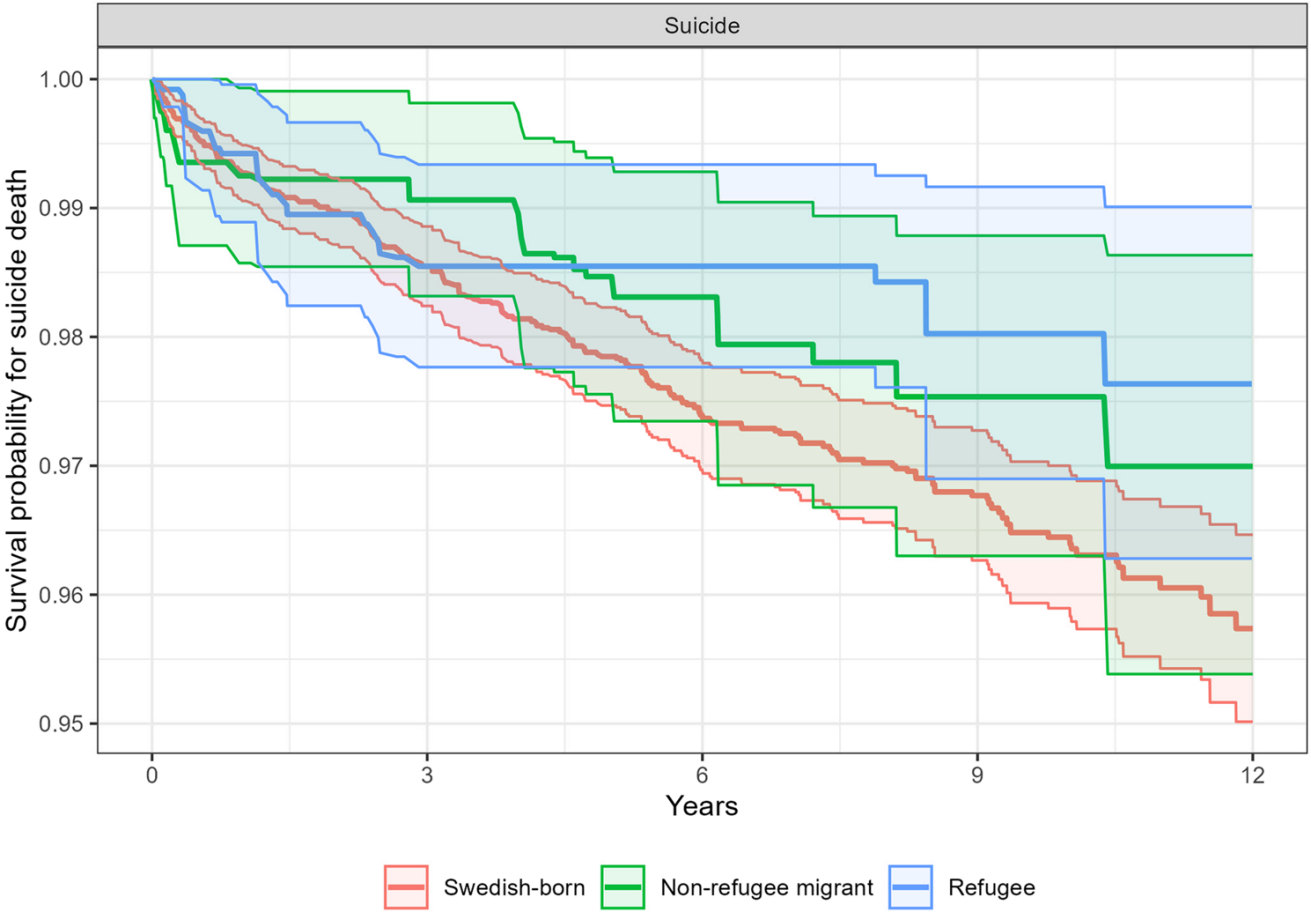
Kaplan-Meier estimated survival curves, incorporating inverse probability weights, for deaths due to suicide during the 5–13-year follow-up period among Swedish-born, non-refugee migrant, and refugee groups with non-affective psychotic disorders

A total of 407 individuals with NAPDs died during the follow-up period, of which 231 (56.8%) were attributable suicide. In the weighted sample, all-cause mortality and suicide deaths were highest among Swedish-born individuals (1.86 and 1.04 per 100,000, respectively) and lowest in the refugee group (1.11 and 0.68 per 100,000, respectively). Cox proportional hazards models (Table 2) indicated that refugees were at significantly lower risk of all-cause mortality (HR = 0.59; 95% CI = 0.39 to 0.91) and suicide (HR = 0.57; 95% CI = 0.34 to 0.96) during the follow-up when compared to Swedish-born individuals (Figs. 2 and 3). Lower risks of all-cause mortality and suicide were also observed for non-refugee migrants relative to Swedish-born individuals, but differences were not statistically significant for either outcome.

## Discussion

In this prospective study of 7,733 young adults with incident NAPDs, we observed that refugees and migrants were at greater risk of developing type 2 diabetes compared to their native-born peers, with refugees also being more likely to experience infectious diseases. In contrast, rates of all-cause mortality and suicide during the 5- to 13-year follow-up period were significantly lower among refugees with NAPDs than in Swedish-born individuals. Whilst our analysis strategy (incorporating IPWs) means that we can be confident that these results are not attributable to the differences in sociodemographic and clinical characteristics that we observed across population groups at baseline, residual confounding may still be present.

To our knowledge, this is the first study to examine associations between migrant/refugee status and physical disorders among individuals with psychotic disorders. In line with our hypotheses, we observed higher rates of specific physical disorders among both migrant groups compared to their native-born peers. Whilst studies in the general population have tended to observe lower rates of some physical disorders among migrants, this is not true of all disorders. Indeed, our finding that non-refugee migrants, and refugees especially, were at greater risk of developing type 2 diabetes is consistent with meta-analytic evidence showing that ethnic minority groups residing in Europe are at greater risk of type 2 diabetes [45]. A range of pre-migration factors (e.g., genetic risk, intrauterine growth, region of origin, socioeconomic status, nutritional intake in early life, trauma) and post-migration factors (e.g., host country, dietary changes, healthcare access, migration reason, psychosocial stress, labour market marginalisation) have been hypothesised underlie the increased risk of type 2 diabetes among migrants within the general population [46]. Many of these risk factors (particularly trauma and psychosocial stress) are likely to be more prevalent among refugees, which might explain why we observed that the elevation in risk was even greater among refugees than for non-refugee migrants. Among those with NAPDs, use of antipsychotic medication may further exacerbate these risks. As such, these findings are highly relevant to clinical teams, as some of these risk factors (including the choice of specific antipsychotic medications associated with weight gain) are potentially modifiable. A further implication of our findings is that refugees and migrants should be monitored for early signs of diabetes and prioritised for behavioural interventions to improve health outcomes. Importantly, a recent study in Norway showing that migrants with type 2 diabetes were less likely to participate in diabetes education program than native-born individuals [47], suggests that increased efforts may be needed to engage migrant groups in such interventions.

The increased risk of infectious diseases that we observed among refugees with NAPDs is a novel and important finding. Infectious diseases (particularly tuberculosis and hepatitis B) are highly prevalent among refugees and asylum seekers [48]. However, as most previous studies have assessed infections at, or shortly after, arrival, poor living conditions during and after migration may account for the increased risk in these populations. The fact that we observed elevated rates of infectious diseases among refugees with NAPDs who had resided in Sweden for at least 3 years (and in most cases, more than 11 years) is therefore particularly striking. Moreover, given that we used diagnoses received in inpatient and specialist outpatient settings, these cases can be considered severe infections. Whilst we accounted for a range of sociodemographic factors that might explain the differences in infection risk between Swedish-born and refugee groups, we were unable to account for living conditions (e.g., number of household residents and environmental exposures), that could feasibly contribute to this finding. Further research is needed to determine the factors that contribute to increased risk in of infections among refugees with NAPDs and to examine the types of infections to which they are most susceptible.

The reduced risk of all-cause mortality that we observed among refugees with NAPDs is consistent with studies conducted in the general population [20]. We are aware of only one previous study comparing all-cause mortality among refugees and migrants [27], in contrast to the present investigation, this study found that refugees with psychotic disorders showed higher all-cause mortality rates (albeit not significantly) than their native-born Swedish-born peers. However, there are some notable differences in study design: individuals in the present study were required to have been resident in Sweden for a minimum of 3 years prior to inclusion and our analysis strategy accounted for a broader range of potential confounders. The former point is particularly pertinent given that previous studies have shown that the mortality advantage observed among migrants within the general Swedish population is dependent on time since arrival and age at migration [49]. Moreover, these same factors appear to modify the risks of treatment for psychotic disorder among migrants [50]. With regard to suicide deaths specifically, our findings are in line with previous studies from Sweden, Norway, and Denmark which have observed lower suicide rates among refugees in the general population compared with their native-born peers [29, 51, 52]. However, in contrast to a study conducted in the Netherlands, we did not observe significantly lower rates of suicide among non-refugee migrants with NAPD compared to native-born individuals with NAPD [28]. One possible explanation for why we did not see the same pattern across refugee and non-refugee migrants is that these groups showed substantial differences in terms of region of birth (Supplementary Table 2), with refugees being more likely to be born in Africa and West Asia. Cultural and religious norms, resilience, and social connectedness (which are likely to be closely tied to region of birth) may act as protective factors in refugees.

### Strengths and limitations

Our study has several strengths, including the use of high-quality data derived from national registers (enabling us to minimise attrition and measure multiple sociodemographic and clinical covariates), a large sample size, and the use of statistical analyses (Cox proportional hazard models incorporating IPWs) that allowed us to remove population group differences in potential confounders. However, our use of register data means that we are unable to capture several factors (e.g., living conditions, family history, genetic factors, diet, and exercise) that might explain the increased risk of specific physical disorders that we observed among refugees and non-refugee migrants. In addition, as sociodemographic and clinical covariates were measured at baseline, we did not account for risk factors accumulating during the follow-up period (e.g., exposure to antipsychotic medication). A further limitation relates to the time period of the study, which included follow-up until the end of 2018. Although more recent data were available at the time at which we undertook these analyses, we chose not to include these additional years due to concerns that the COVID-19 pandemic may affect rates of our outcomes of interest (i.e., infectious diseases and all-cause mortality) and that migrant groups would be disproportionality affected by these effects (potentially leading to bias). Finally, our findings may have limited generalisability to countries without publicly financed healthcare systems.

## Conclusions

The number of refugees worldwide has risen exponentially, increasing from 25.7 million to 32 million during the first half of 2022 alone [53], a trend which can be expected to continue given recent global and political events. These global changes have important implications for healthcare services, which will need to adapt, and become more culturally competent, in order to meet the needs of these vulnerable groups. This is particularly relevant for psychiatric services, especially those which care for individuals with psychotic disorders, given the over-representation of refugee and non-refugee migrant groups in this population: Indeed, consistent with our previous work using data from Sweden and Denmark [54], refugee and non-refugee migrant groups formed nearly 25% of the NAPD population in the current study. Our findings suggest that interventions to improve physical health among individuals with NAPDs should be specifically targeted at migrant groups, particularly refugees, who we have shown here to be at increased risk of developing type 2 diabetes and infectious diseases. Further research is needed to determine the potentially modifiable factors that contribute to the excess physical morbidity among migrants NAPDs and to investigate differences in cause-specific mortality rates.

## Supplementary Information

Supplementary Material 1.

## Data Availability

These data cannot be made publicly available due to privacy regulations. According to the General Data Protection Regulation, the Swedish law SFS 2018:218, the Swedish Data Protection Act, the Swedish Ethical Review Act, and the Public Access to Information and Secrecy Act, these types of sensitive data can only be made available for specific purposes, including research, that meets the criteria for access to this type of sensitive and confidential data as determined by a legal review. Readers may contact Professor Kristina Alexanderson (kristina.alexanderson@ki.se) regarding the data.

## Abbreviations

ATC: Anatomical Therapeutic Chemical
CI: Confidence interval
HR: Hazard ratios
ICD-10: International Classification of Diseases– version 10
IPW: Inverse probability weighting
IQR: Interquartile range
LISA: Longitudinal Integrated Database for Health Insurance and Labour Market Studies
MiDAS: Micro-Data for Analyses of Social Insurance
NAPD: Non-affective psychotic disorder
NPR: National Patient Register
PDR: Prescribed Drug Register
STATIV: Longitudinal Database for Integration Studies
STROBE: Strengthening the Reporting of Observational Studies in Epidemiology

## Footnotes

1 Asthma was not included as a respiratory disorder as our interest was in incident physical disorders (i.e., occurring after NAPD onset) and asthma typically onsets in childhood.
