# Supplementary material for: Associations of migrant and refugee status with physical comorbidity and mortality among young adults with incident non-affective psychotic disorders
Source: BMC Public Health. 2025 Aug 6;25:2680. doi: 10.1186/s12889-025-24054-8 (PMC12326835; doi:10.1186/s12889-025-24054-8)
Supplement: Supplementary file 1 — Supplementary Material 1. [file 12889_2025_24054_MOESM1_ESM.docx]

Supplementary Material

Cullen AE, de Montgomery CJ, Pettersson E, Akhtar A, Norredam M, Taipale H, Mittendorfer-Rutz E. Impact of migrant and refugee status on physical comorbidity and mortality among young adults with incident non-affective psychotic disorders.

Supplementary Table 1. Follow-up time (years) during the study period by population group

Supplementary Table 2. Region of birth and duration of residence for non-refugee migrant and refugee populations

Supplementary Table 3. Maximum values of weighted standardized mean differences (continuous variables) and weighted proportion differences (binary variables) for all covariates across cardiovascular disease, type 2 diabetes, infectious diseases, respiratory diseases, all-cause mortality outcomes

**Supplementary Table 1.** Follow-up time (years) during the study period by population group

|  | Total cohort  (*n =* 7,733 100%) | | Swedish-born  (*n =* 5,870, 75.9%) | | Non-refugee migrant  (*n =* 861, 11.1%) | | Refugee  (*n =* 1,002, 13.0%) | |
| --- | --- | --- | --- | --- | --- | --- | --- | --- |
|  |  |  |  |  |  |  |  |  |
| Mean (S.D.) | 8.6 | (2.8) | 8.6 | (2.7) | 8.4 | 3.1 | 8.5 | 2.8 |
| Median (IQR) | 8.7 | (6.5–10.9) | 8.7 | (6.6–10.9) | 8.4 | 6.2–11.0 | 8.7 | 6.5–10.7 |
| Min, max | 5.0 | 13.0 | 5.0 | 13.0 | 5.0 | 13.0 | 5.0 | 13.0 |

IQR, interquartile range.

**Supplementary Table 2.** Region of birth and duration of residence for non-refugee migrant and refugee groups

|  | Non-refugee migrant (*n* = 861) | | Refugee  (*n* = 1,002) | |
| --- | --- | --- | --- | --- |
| **Region of birth** |  | |  | |
| Africa | 125 | (14.5) | 236 | (23.6) |
| Asia | 216 | (25.1) | 143 | (14.3) |
| Europe | 304 | (35.3) | 342 | (34.2) |
| Other | 123 | (14.3) | 38 | (3.8) |
| West Asia | 93 | (10.8) | 243 | (24.3) |
| **Duration of residence** |  |  |  |  |
| 0-5 years | 128 | (15.6) | 108 | (10.8) |
| 6-10 years | 210 | (25.7) | 185 | (18.5) |
| 11+ years | 480 | (58.7) | 709 | (70.8) |

Duration of residence data missing for 43 non-refugee migrants.

**Supplementary Table 3.** Maximum values of weighted standardized mean differences (continuous variables) and weighted proportion differences (binary variables) across all exposure pairs

|  | Cardiovascular disease | | Type 2 diabetes | | Infectious disease | | Respiratory disease | | All-cause mortality and suicide | |
| --- | --- | --- | --- | --- | --- | --- | --- | --- | --- | --- |
|  | Crude | Weighted | Crude | Weighted | Crude | Weighted | Crude | Weighted | Crude | Weighted |
| Age | 0.5153 | 0.0001 | 0.5249 | 0.0001 | 0.5135 | 0.0001 | 0.5128 | 0.0001 | 0.5161 | 0.0001 |
| Gender (female) | 0.1573 | <0.0001 | 0.1565 | <0.0001 | 0.1658 | <0.0001 | 0.1595 | <0.0001 | 0.1586 | <0.0001 |
| Education (compulsory) | 0.1711 | <0.0001 | 0.1699 | <0.0001 | 0.1791 | <0.0001 | 0.1726 | <0.0001 | 0.1709 | <0.0001 |
| Education (high school) | 0.1032 | <0.0001 | 0.1042 | <0.0001 | 0.0912 | <0.0001 | 0.1038 | <0.0001 | 0.1052 | <0.0001 |
| Education (university) | 0.1242 | 0.0001 | 0.1261 | 0.0001 | 0.1328 | <0.0001 | 0.1260 | 0.0001 | 0.1263 | 0.0001 |
| Family situation (married/cohabiting) | 0.1676 | <0.0001 | 0.1694 | <0.0001 | 0.1630 | <0.0001 | 0.1660 | <0.0001 | 0.1694 | <0.0001 |
| Residence region (cities) | 0.1490 | <0.0001 | 0.1503 | <0.0001 | 0.1444 | <0.0001 | 0.1483 | <0.0001 | 0.1499 | <0.0001 |
| Residence region (towns/suburbs) | 0.0748 | 0.0001 | 0.0764 | 0.0001 | 0.0693 | 0.0001 | 0.0736 | 0.0001 | 0.0756 | 0.0001 |
| Residence region (rural areas) | 0.0742 | 0.0001 | 0.0739 | 0.0001 | 0.0751 | 0.0001 | 0.0747 | 0.0001 | 0.0744 | 0.0001 |
| Household income (< 60 pct of median) | 0.1618 | <0.0001 | 0.1626 | <0.0001 | 0.1721 | <0.0001 | 0.1654 | <0.0001 | 0.1632 | <0.0001 |
| Unemployment days (any) | 0.1380 | <0.0001 | 0.1367 | <0.0001 | 0.1297 | <0.0001 | 0.1370 | <0.0001 | 0.1365 | <0.0001 |
| Sickness absence days (>30) | 0.0276 | <0.0001 | 0.0287 | <0.0001 | 0.0265 | <0.0001 | 0.0298 | <0.0001 | 0.0289 | <0.0001 |
| Disability pension (any) | 0.0465 | <0.0001 | 0.0460 | 0.0001 | 0.0417 | <0.0001 | 0.0449 | <0.0001 | 0.0460 | <0.0001 |
| Cohort entry year | 0.0562 | <0.0001 | 0.0587 | <0.0001 | 0.0684 | <0.0001 | 0.0599 | <0.0001 | 0.0586 | <0.0001 |
| NAPD diagnosis (schizophrenia) | 0.0250 | 0.0001 | 0.0249 | 0.0001 | 0.0337 | 0.0001 | 0.0251 | 0.0001 | 0.0250 | 0.0001 |
| NAPD diagnosis (schizotypal) | 0.0200 | <0.0001 | 0.0198 | <0.0001 | 0.0203 | <0.0001 | 0.0200 | <0.0001 | 0.0200 | <0.0001 |
| NAPD diagnosis (delusional disorder) | 0.0096 | 0.0001 | 0.0098 | 0.0001 | 0.0176 | <0.0001 | 0.0101 | 0.0001 | 0.0092 | 0.0001 |
| NAPD diagnosis (acute or transient) | 0.0303 | 0.0001 | 0.0316 | 0.0001 | 0.0204 | <0.0001 | 0.0333 | 0.0001 | 0.0309 | 0.0001 |
| NAPD diagnosis (other) | 0.0281 | <0.0001 | 0.0252 | <0.0001 | 0.0107 | <0.0001 | 0.0268 | <0.0001 | 0.0253 | <0.0001 |
| Prior psychiatric disorder (any) | 0.1111 | <0.0001 | 0.1094 | <0.0001 | 0.1034 | <0.0001 | 0.1094 | <0.0001 | 0.1101 | <0.0001 |
| Prior physical condition (any) | 0.0247 | <0.0001 | 0.0241 | <0.0001 | 0.0360 | <0.0001 | 0.0238 | <0.0001 | 0.0232 | <0.0001 |
| Prior suicide attempt (any) | 0.0267 | 0.0001 | 0.0271 | 0.0001 | 0.0223 | <0.0001 | 0.0252 | 0.0001 | 0.0263 | 0.0001 |
| Psychotropic medication (any) | 0.1312 | <0.0001 | 0.1328 | <0.0001 | 0.1267 | <0.0001 | 0.1293 | <0.0001 | 0.1319 | <0.0001 |

NAPD, non-affective psychotic disorder.
